# B- and N-doped carbon dots by one-step microwave hydrothermal synthesis: tracking yeast status and imaging mechanism

**DOI:** 10.1186/s12951-021-01211-w

**Published:** 2021-12-28

**Authors:** Bo Tian, Tianxin Fu, Yang Wan, Yun Ma, Yanbo Wang, Zhibiao Feng, Zhanmei Jiang

**Affiliations:** 1 College of Food Science, Northeast Agricultural University, Harbin, 150030 China; 2 Department of Chemistry, Northeast Agricultural University, Harbin, 150030 China

**Keywords:** BN-CDs, Yeast, Imaging, Different lethal modes, Endocytosis

## Abstract

**Background:**

Carbon dots (CDs) are widely used in cell imaging due to their excellent optical properties, biocompatibility and low toxicity. At present, most of the research on CDs focuses on biomedical application, while there are few studies on the application of microbial imaging.

**Results:**

In this study, B- and N-doped carbon dots (BN-CDs) were prepared from citric acid, ethylenediamine, and boric acid by microwave hydrothermal method. Based on BN-CDs labeling yeast, the dead or living of yeast cell could be quickly identified, and their growth status could also be clearly observed. In order to further observe the morphology of yeast cell under different lethal methods, six methods were used to kill the cells and then used BN-CDs to label the cells for imaging. More remarkably, imaging of yeast cell with ultrasound and antibiotics was significantly different from other imaging due to the overflow of cell contents. In addition, the endocytosis mechanism of BN-CDs was investigated. The cellular uptake of BN-CDs is dose, time and partially energy-dependent along with the involvement of passive diffusion. The main mechanism of endocytosis is caveolae-mediated.

**Conclusion:**

BN-CDs can be used for long-term stable imaging of yeast, and the study provides basic research for applying CDs to microbiol imaging.

**Graphical Abstract:**

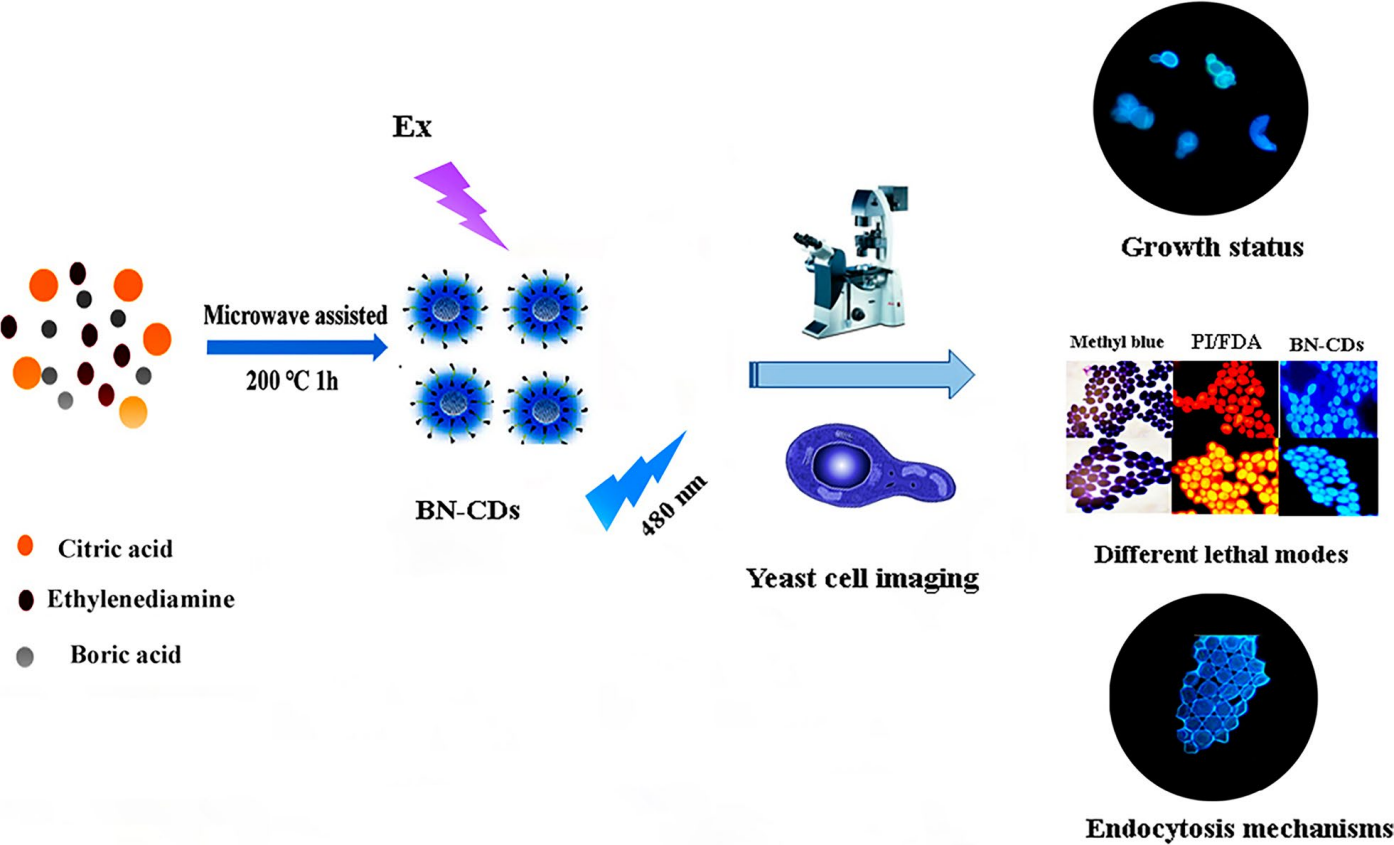

**Supplementary Information:**

The online version contains supplementary material available at 10.1186/s12951-021-01211-w.

## Background

As an emerging field of research, nanotechnology is incessantly carving its own forte. Nanomaterials have been widely used in food packaging [1], probiotics encapsulating [2, 3], antibacterial [4], drug delivery [5] and biosensing [6]. CDs are a new type of zero-dimensional nanomaterials, with a particle diameter of less than 10 nm, and nano-carbon particles with fluorescent properties [7]. Due to the advantages of high fluorescence intensity [8], low toxicity [9], good biocompatibility [10] and easy modification [11], CDs have attracted extensive attention. There are a wide range of materials for the synthesis of CDs, among which the most classic precursors are citric acid and ethylenediamine developed by Zhu [12]. These CDs have stable fluorescence properties, high fluorescence quantum yield and easy to be modified. The surface of CDs can be functionalized by doping heteroatoms to improve the quantum yield and property [13]. The common doping elements include phosphorus [14], sulfur [15], nitrogen [16] and boron [17]. In recent years, more and more attention has been paid to boron. Since the electronegativity of boron element is lower than that of C atoms, the B atoms doped in CDs can form a positive charge distribution around the C atoms [19]. Due to electrostatic adsorption, this change in charge is conducive to the adsorption of B-CDs and biomolecules, which is conducive to cell imaging [20].

At present, there are many reports on the imaging of CDs in animal cells [20–25]. In contrast, there are few studies on the imaging of CDs in microorganisms, especially the mechanism of microbial uptake of CDs and the distribution of CDs in cells. Paul et al. used a one-step hydrothermal method to produce gelatin quantum dots that could be stably combined with *E. coli*, *S. aureus*, *C. albicans*, *C. krusei*, *C. parapsilosis*, *and C. tropicalis* causing the cells to emit green, red, and blue at different excitation wavelength [26]. Ji et al. used *Weissella *sp. to make CDs-Ws, which could label dead bacteria and dead yeast. For dead cell, they thought the charge on the surface changed, which made the cell wall easier to bond with CDs-Ws [27]. The cell wall and membrane of the dead microbial cell was destroyed,so CDs-Ws could more easily enter the inside of the microbial cell. In addition, sucrose juice could be used to synthesize CDs, which could enter *E.coli* and *S.cerevisiae* after 6 h of co-incubation [28]. CDs synthesized from fresh tomato pulp were used for biological imaging of plant pathogenic fungi [29]. Moreover, imaging studies of *B. subtilis*, *A. aculeatus* [30], *A. flavus*, *A. fumigatus* [31], *M. tuberculosis* and *P. aeruginosa* [32], and *S. cerevisiae* [33] *F. avenaceum* [34] were also carried out based on CDs. The current research on microbial imaging based on CDs mainly focuses on the preparation and structural characterization of CDs [34–37], and whether the CDs can be used for microbial labeling [37–42]. The observation and analysis of the growth status of microorganisms using CDs are rare, and the mechanism of microbial cell uptake of CDs is still not clear.

Cellular internalization of nanoparticles depends not only on size, surface charge, shape, and surface modification of the nanoparticles, but also on cell types [42]. After the uptake of nanoparticles by cells, they interact with different subcellular components and organelles, leading to their delivery to different intracellular organelles, which is directly related to the cytotoxicity or biological function of the internalized nanoparticles [43]. Therefore, the exact endocytosis mechanism and intracellular localization of CDs are essential for assessing their biological properties and improving further understanding of their growth status.

In this work, we developed a one-step microwave hydrothermal method for the preparation of BN-CDs with good fluorescence properties. The application of BN-CDs in yeast imaging was studied. The prepared BN-CDs showed a good potential in the identification of yeast viability and yeast growth status. The mechanism of BN-CDs uptake by yeast was also studied. The study provides basic research for the better application of CDs in yeast imaging.

## Results and discussion

### Preparation and characterization of BN-CDs

Our group previously reported that BN-CDs with high quantum yield and good fluorescence stability were synthesized by hydrothermal method, and it had been successfully applied to microbial labeling. In this study, BN-CDs were synthesized by microwave hydrothermal method. The advantage of microwave hydrothermal method is that it can shorten the time of CDs synthesis, and the size of CDs is more uniform and smaller, which is more conducive to microbial imaging [13].

**Fig. 1:**
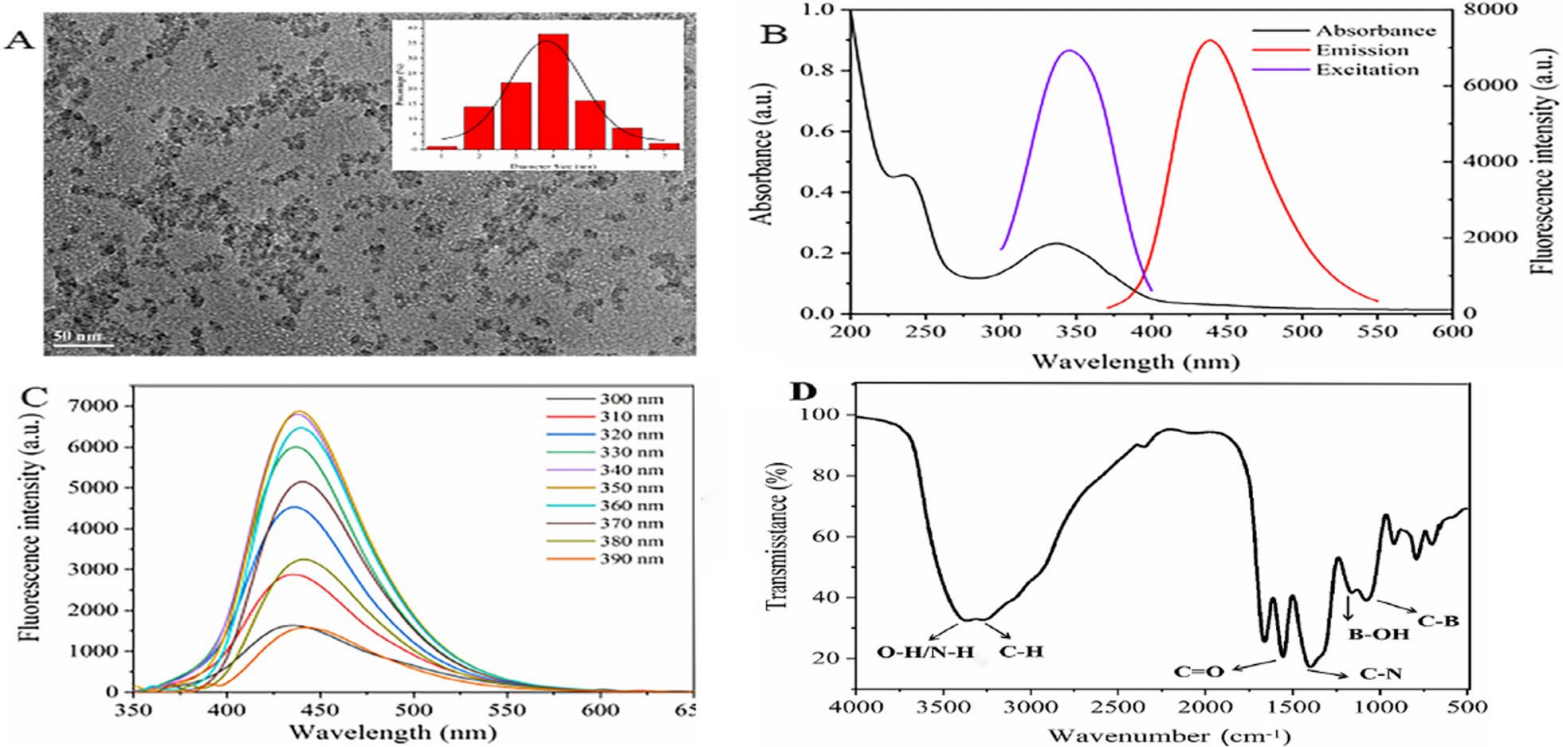
Synthesis and characterization of BN-CDs. **A **TEM image and particle size distribution. **B** Ultraviolet spectrogram. **C** Fluorescence spectra. **D** Fourier transform infrared spectrogram

The morphology of BN-CDs was characterized using the transmission electron microscopy (TEM). As shown in Fig. 1A, BN-CDs were uniformly dispersed, and the particle diameter was concentrated around 4 nm. In the UV spectrum (Fig. 1B), there were two absorption bands at 238 nm and 336 nm, which was due to the *π* = π* electronic transition of C = C and the n–π* transition of surface groups (C = O and C = N) [41]. In addition, BN-CDs possessed optimal excitation and emission wavelengths at 350 nm and 446 nm (Fig. 1C). As shown in Fig. 1D, an intense and broad peak at about 3350 cm^−1^ was attributed to stretching vibration such as O–H/N–H [44], and stretching vibrations of C–H (3250 cm^−1^), C = O (1654 cm^−1^) [45] bonds were observed. The peak at 1554 cm^−1^ corresponded to C–N stretching vibration, which indicated that N atom has been successfully introduced into BN-CDs as passivator [46]. The peak at 1165 cm^−1^ corresponded to the C-B vibration, indicating that B atom was successfully doped into BN-CDs [47].

**Fig. 2:**
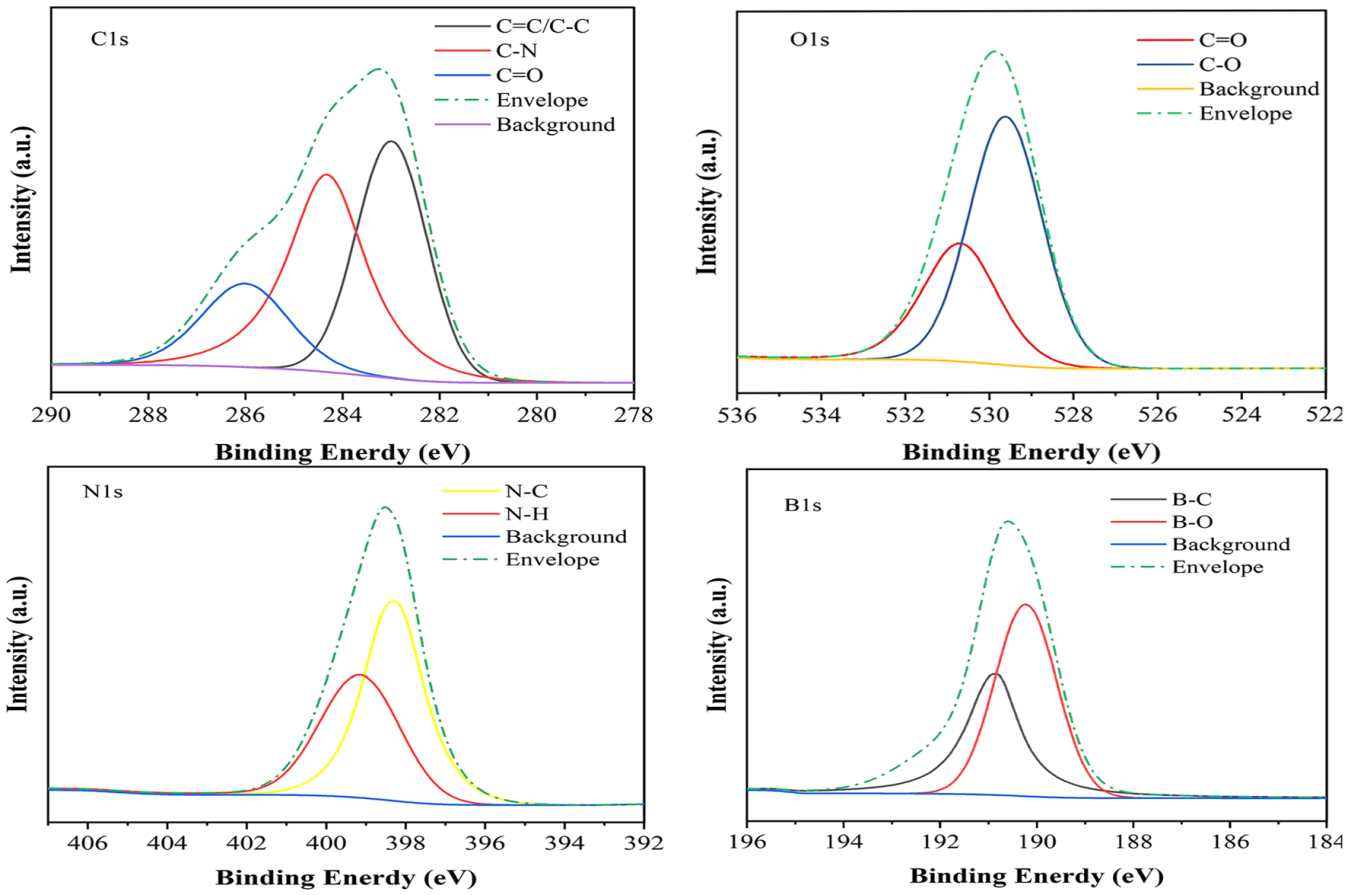
High-resolution XPS C 1 s, N 1 s, and O 1 s spectra of BN-CDs

X-ray photoelectron spectroscopy (XPS) was used to further study the surfaces of BN-CDs. The full spectrum (Additional file 1: Fig. S2) showed four typical peaks: B 1 s, C 1 s, N 1 s and O 1 s. In the high-resolution spectrum (Fig. 2), the C 1 s band was convoluted into three peaks, corresponding to sp^2^ carbon (C = C, 283.5 eV), sp^3^ carbon (C–N, 284.35 eV) and carbonyl carbon (C = O, 286.1 eV), respectively [48]. The N 1 s band was convoluted into two peaks at 399.1 and 398.35 eV, representing N–H and N–C, respectively. O 1 s XPS spectrum was decomposed into peaks at 530.6 and 529.5 eV, corresponding to O = C and O–C, respectively [49]. The spectrum of B 1 s exhibited two peaks at 190.8 and 190.2 eV, which could be assigned to B–C and B–O, respectively [42]. The fluorescence quantum yields (QYs) of BN-CDs was calculated to be 66.59% using quinine sulfate as a standard. And their fluorescence lifetime was 125.63 ns (Additional file 1: Fig. S1), indicating that BN-CDs have better fluorescence stability.

Turbidity method is a conventional method to detect the growth of bacteria according to the turbidity of bacteria suspension. In order to determine whether BN-CDs can be used as probes for yeast imaging, we used the turbidimetric method to detect the toxicity of BN-CDs to yeast. It was found that when the concentration of BN-CDs reached 400 mg/mL, the growth curve of yeast was not significantly affected, which indicated that the cytotoxicity of BN-CDs was low (Additional file 1: Fig. S3), and confirmed that BN-CDs could act as eco-friendly biological fluorescent-labeling probes for yeast imaging.

**Fig. 3:**
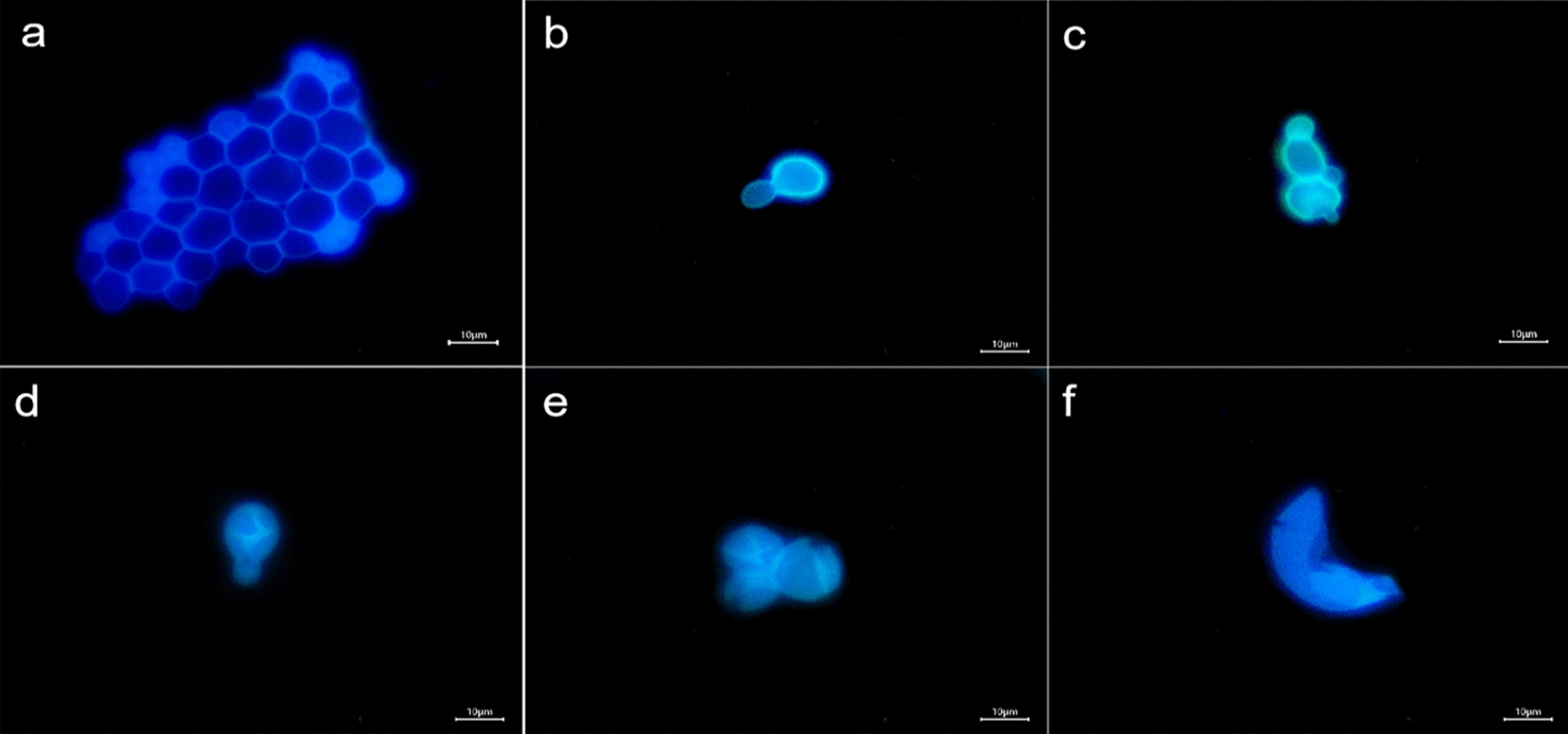
Fluorescence microscope images of yeast in different growth status. **a** Living and dead yeast cells. **b **Yeast cell budding. **c** Multiple budding of yeast cell. **d** Ascomycetes of yeast cell. **e** Ascospores of yeast cell. **f** Rupture of aging yeast cell. (Scale bar  = 10 μm)

Yeast can be clearly labeled with BN-CDs in only 1 min.(Fig. 3) Further observation showed that there were two status in the image of yeast cell, one was bright blue, and the other was dark blue with a bright halo, which was related to the life and death of yeast cell [50] (Fig. 3a). Yeast cell wall has a certain thickness, and cell membrane also has selective permeability, so BN-CDs was difficult to enter the living yeast cell in a short time. As a result, the fluorescence in living yeast cell was weak and dark blue. For dead yeast cell, the structure of protein and phospholipid bilayer in cell membrane is destroyed, resulting in the increase of cell permeability. Therefore, BN-CDs could quickly enter the cell, making the whole cell bright blue.

The budding pattern of the yeast cell can be clearly observed by using BN-CDs. There are two ways of yeast cell reproduction: sexual reproduction and asexual reproduction. Budding is the most common way of asexual reproduction of yeast [51]. As shown in Fig. 3b, it was the budding status of yeast cell. The surface of yeast cell protruded outward and sprouted. When the buds grew to normal size, they would separate from the mother and become independent cells. Each mature yeast cell could sprout at one or more places (Fig. 3c). According to Fig. 3b, c; a bright blue color appeared inside the yeast cell during budding, indicating that the BN-CDs entered the budding yeast cell. During the budding process of yeast cell, the hydrolase decomposed cell wall polysaccharides to make cell wall thinner, so BN-CDs could enter cell in a short time [52]. In addition to budding, when the nutritional status of yeast cell was not good, some cells could carry out sexual reproduction, formed spores (generally four spores), and germinated when the conditions were suitable (Fig. 3d), while the original vegetative cell became ascospores (Fig. 3e) [53]. However, with the gradual aging of yeast cell, the protein expression was abnormal, and finally part of the aging cell appeared lysis (Fig. 3f). It can be concluded that BN-CDs can label yeast in a short time and clearly observe the status of yeast cell, which plays a positive role in understanding the growth process of yeast. BN-CDs are very different from the fluorescent CDs currently reported [25–28]. For those CDs, it needed a long co-incubation time for labeling yeast, and the growth status of yeast cell could not be observed. For BN-CDs, it only took one minute to label yeast, and the image was not only clear, but also showed the growth stage of yeast cell. BN-CDs staining can be used as a rapid screening method to monitor yeast during the fermentation process, and it is good for adjusting fermentation conditions in time.

### Yeast imaging with different lethal methods

**Fig. 4:**
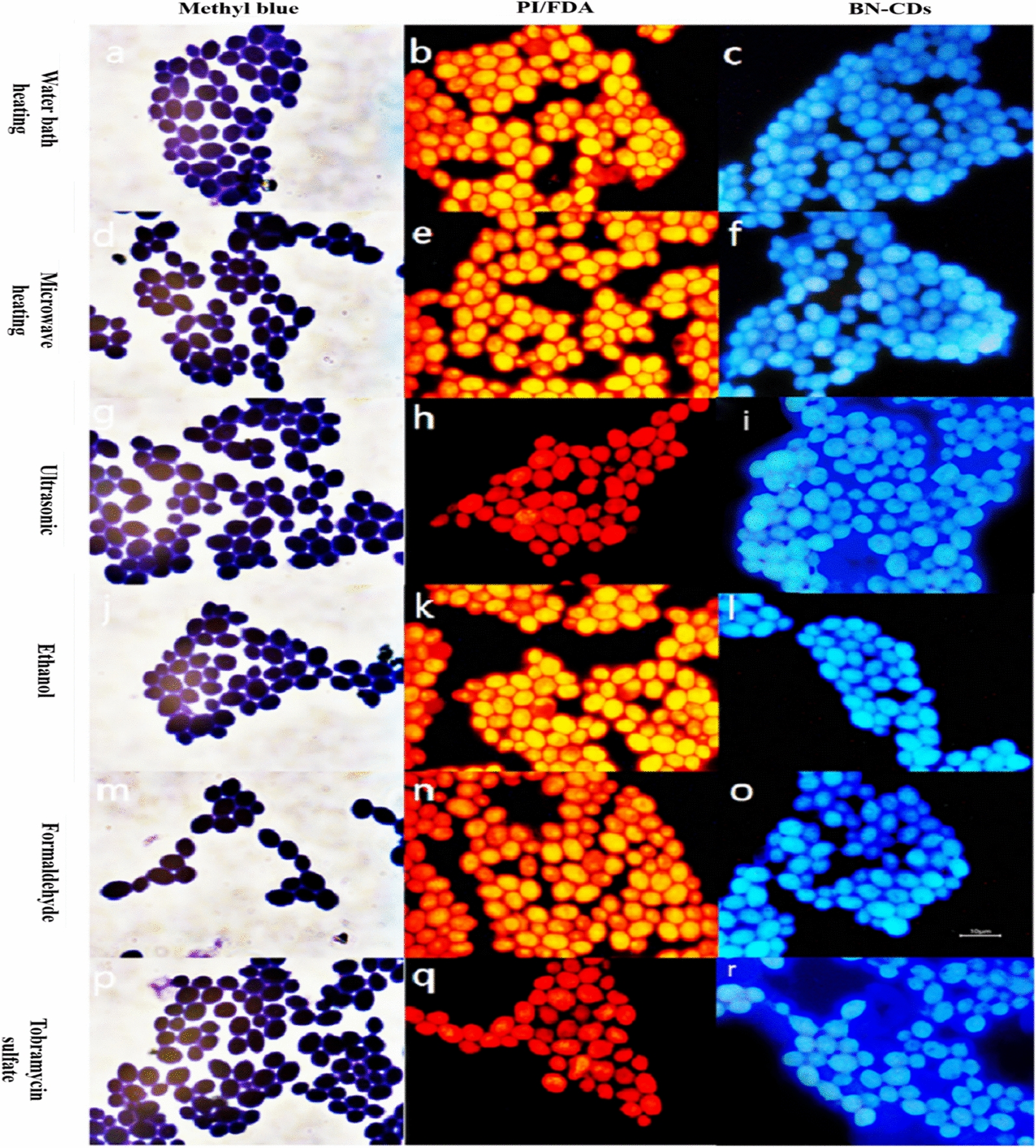
Fluorescence microscope images of yeast with different lethal methods. (Scale bar  = 10 μm)

In order to further confirm that BN-CDs can quickly identify the dead and living yeast cells, six different methods were used to kill yeast. Methyl blue staining and pyridine iodide (PI)/fluorescein diacetate (FDA) staining were used as control. The dehydrogenases in the active yeast cell promote the reduction of methylene blue to a colorless substance, while dead yeast cell remains blue. PI can pass through the dead cell membrane and bind with DNA to emit red light. As shown in Fig. 4, the yeast cells stained with methylene blue were all blue, and those stained with PI/FDA were red, which indicated that they were all dead. Using BN-CDs to label the same yeast sample, as shown in Fig. 3, the whole yeast cell was bright blue, which was significantly different from the image of living yeast cell (Fig. 3a). It further confirmed that BN-CDs can identify the viability of yeast cell, which is consistent with the report of Ma [50].

It was worth noting that there was no obvious difference in the imaging when using methylene blue to stain yeast killed in different ways. However, when PI/FDA and BN-CDs staining were used, the images of cell killed by ultrasound and tobramycin sulfate were significantly different from those of other methods. The cell stained with PI/FDA was darker red, while yeast cell stained with BN-CDs, its surroundings and itself were bright blue. Water bath heating, microwave heating, formaldehyde and ethanol treatment will denature the protein in yeast cell, thereby killing the cells. Using these four methods to kill yeast there is no significant difference in imaging. However, the mechanical shearing force generated by ultrasound breaks cell membrane, which caused the cell contents to overflow. PI staining requires repeated washing, which reduces the binding of PI and cells, resulting in weaker fluorescence. When yeast was stained with BN-CDs, there was no need to wash, so BN-CDs marked the substances leaked out of the cell, causing bright blue light around the cell. Similarly, tobramycin sulfate could increase the permeability of cell membrane, leading to the leakage of potassium ions, adenine nucleotides, enzymes and other important substances in cell, so its image was similar to that of yeast cell killed by ultrasound.

In summary, the advantage of using BN-CDs to stain yeast is that there is no need to repeatedly wash, which avoids the experimental error caused by washing off cells during operation. BN-CDs staining can also quickly identify the death of yeast, and speculate whether the cause of death is related to cell membrane destruction.

### Cellular uptake kinetics

**Fig. 5:**
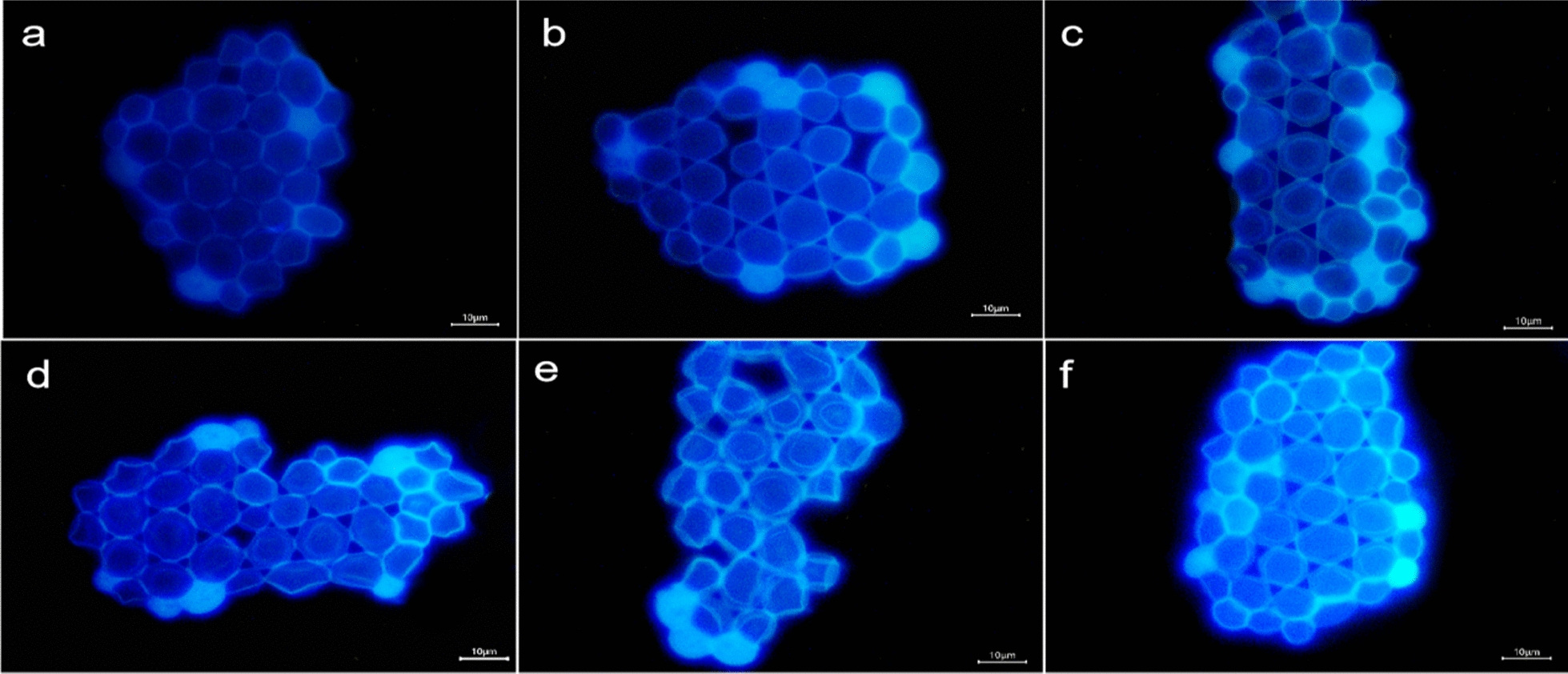
Fluorescence microscope images of yeast co-incubated with BN-CDs at oncentrations 10 (**a**), 50 (**b**), 100 (**c**), 150 (**d**), 200 (**e**) and 300 (**f**) (mg/mL) for 120 min, respectively. (Scale bar  = 10 μm)

**Fig. 6:**
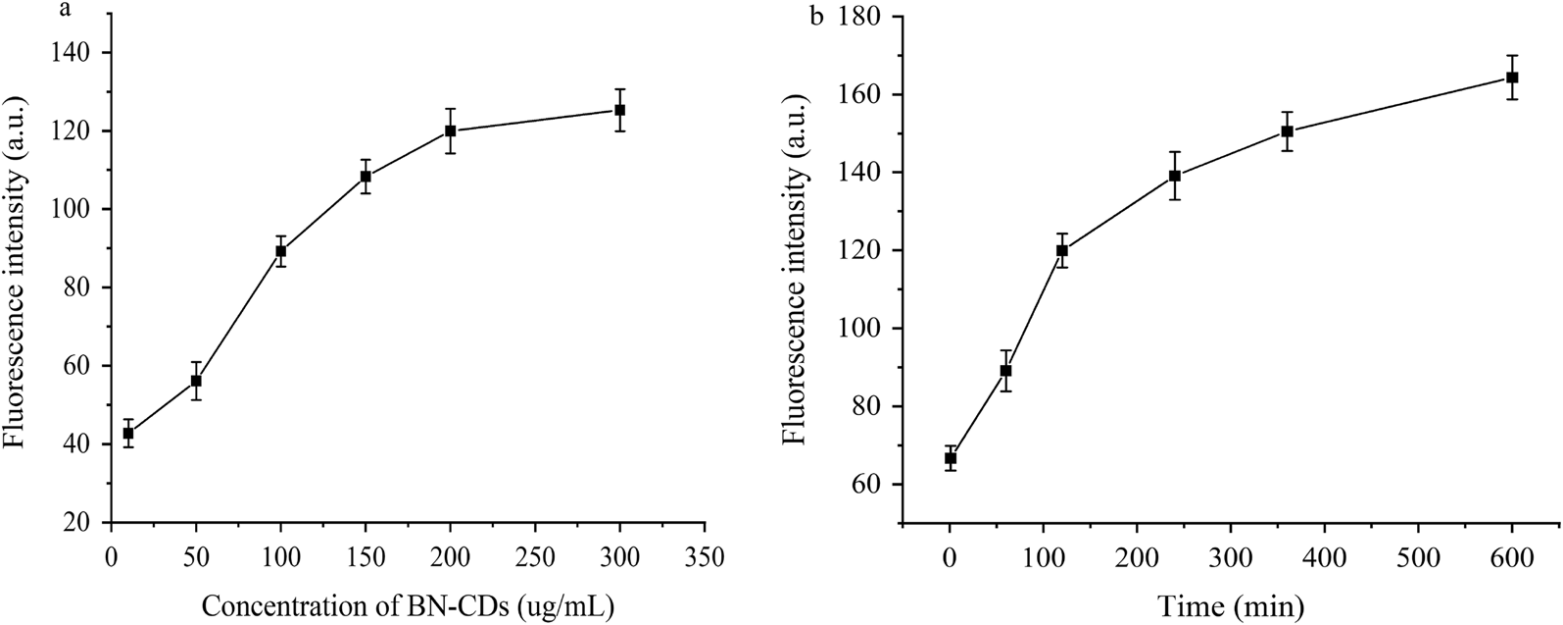
The fluorescence intensity inside yeast cells. **a **Concentration of BN-CDs. **b** Incubation time

**Fig. 7:**
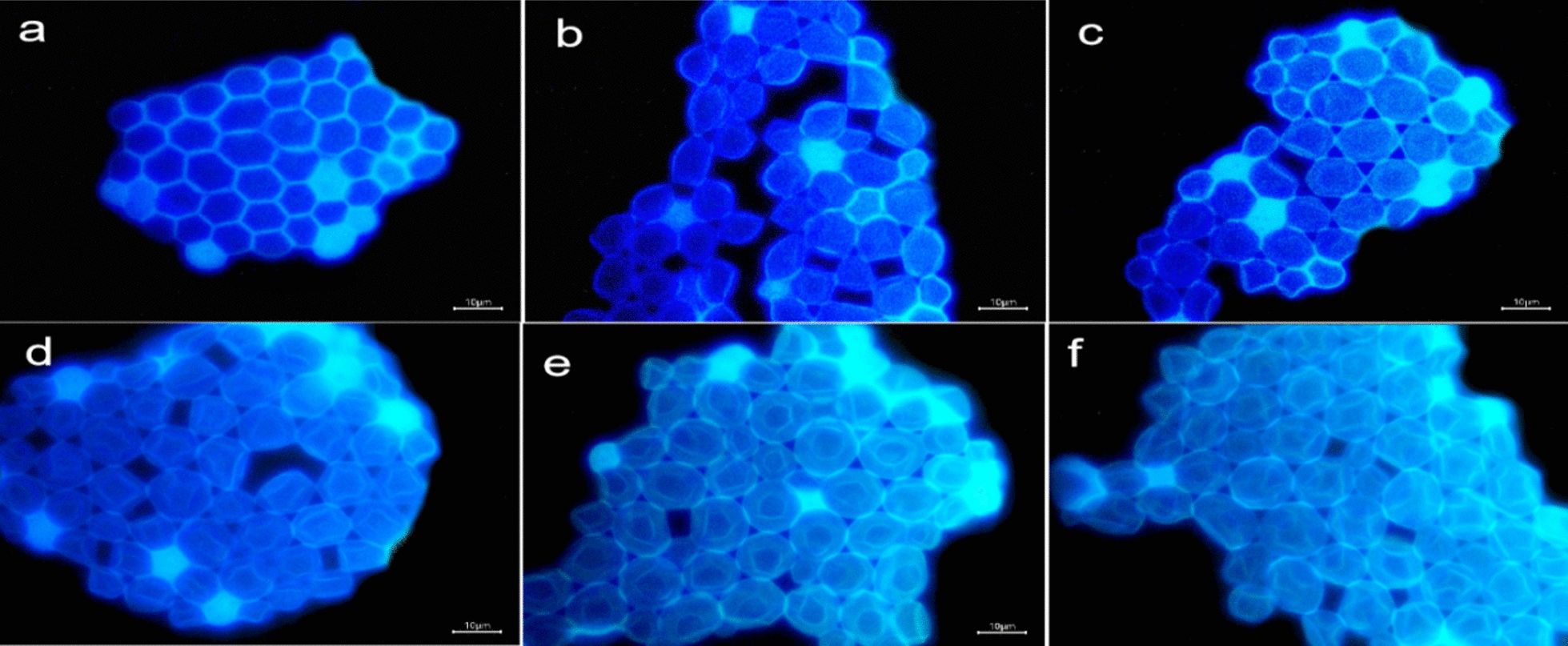
Fluorescence microscope images of yeast co-incubated with BN-CDs for 1 (**a**), 60 (**b**), 120 (**c**), 240 (**d**), 360 (**e**), and **f** 600 min, respectively. (Scale bar  = 10 μm)

It can be found that with the increase of BN-CDs concentration, the imaging of yeast becomes clearer, fluorescence inside the cell is also gradually enhanced (Fig. 5), and the average fluorescence intensity of cell increases significantly (Fig. 6a). All these indicate that the cellular uptake of BN-CDs has a dose-dependent characteristic. As shown in Fig. 5, the image of yeast cell is the clearest when the concentration of BN-CDs is 200 μg/mL, so BN-CDs (200 μg/mL) were used to label yeast in subsequent experiments. According to Fig. 7a, b, yeast was incubated with BN-CDs for a short period of time, the outer wall of some yeast cells had a bright light halo, while the inside was dark blue, and the cell wall and cytoplasm were clearly distinguished. With the extension of co-incubation time of BN-CDs and yeast, the fluorescence intensity in cells gradually increased, and the distribution of BN-CDs within cells became more and more uniform. As shown in Fig. 7, with the increase of incubation time, the fluorescence intensity inside cells became stronger and stronger., which showed that the cellular uptake of BN-CDs was time-dependent.

### Endocytosis pathway of BN-CDs into yeast

Endocytosis, also known as transcytosis, is the process of transporting extracellular material into the cell through the deformation movement of plasma membrane. The endocytosis of nanoparticles is not only related to their size, surface potential, shape and surface chemical modification, but also depends on different cell types [54]. Low-temperature, chlorpromazine and genistein were used to inhibit cell energy-dependent, clathrin-mediated and caveolin-mediated endocytosis, respectively.

**Fig. 8:**
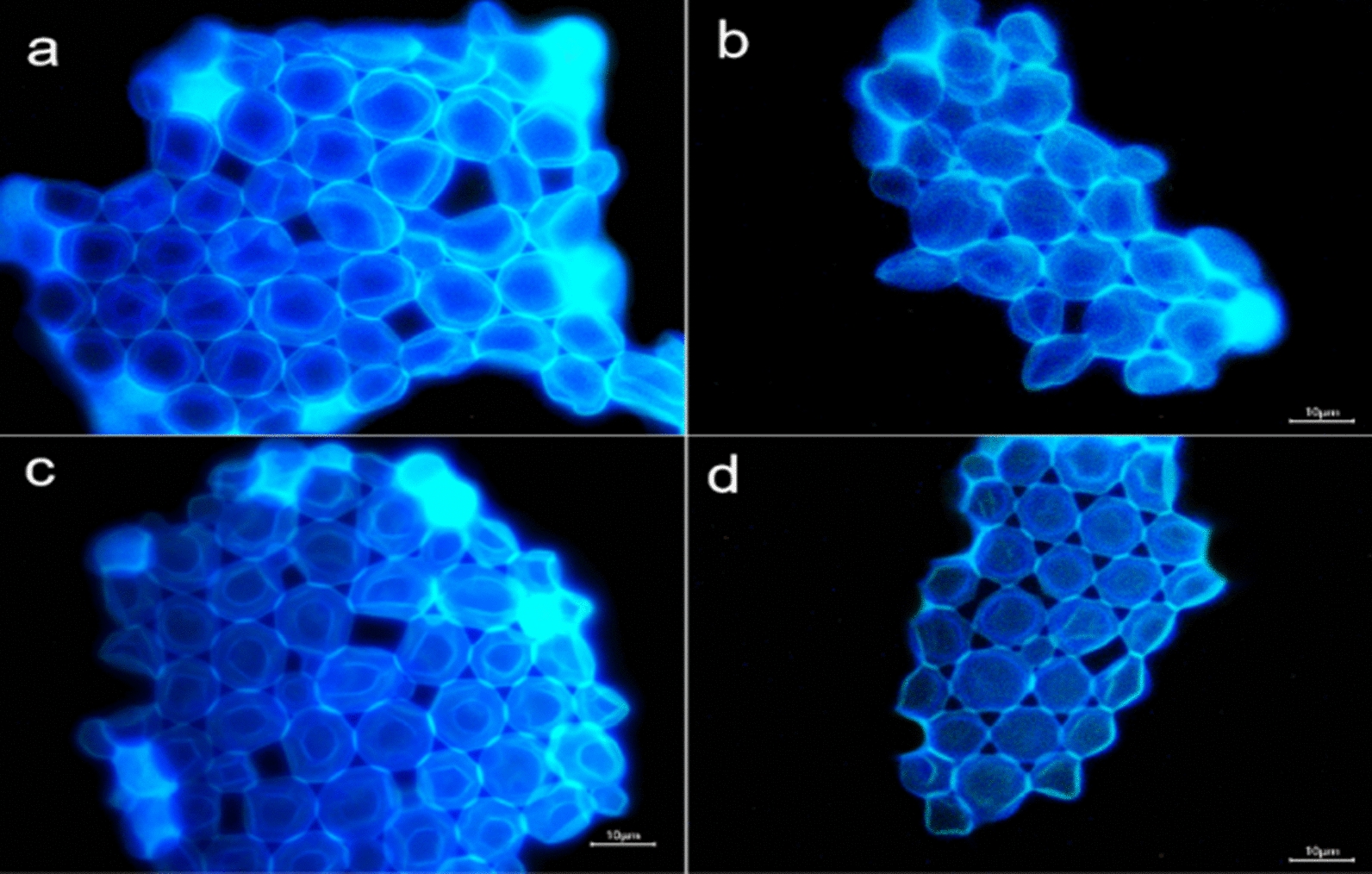
Fluorescence microscope images of yeast stained with BN-CDs. **a** Blank control group, **b** low temperature, **c** Chlorpromazine, **d** genistein. (Scale bar  = 10 μm)

**Fig. 9:**
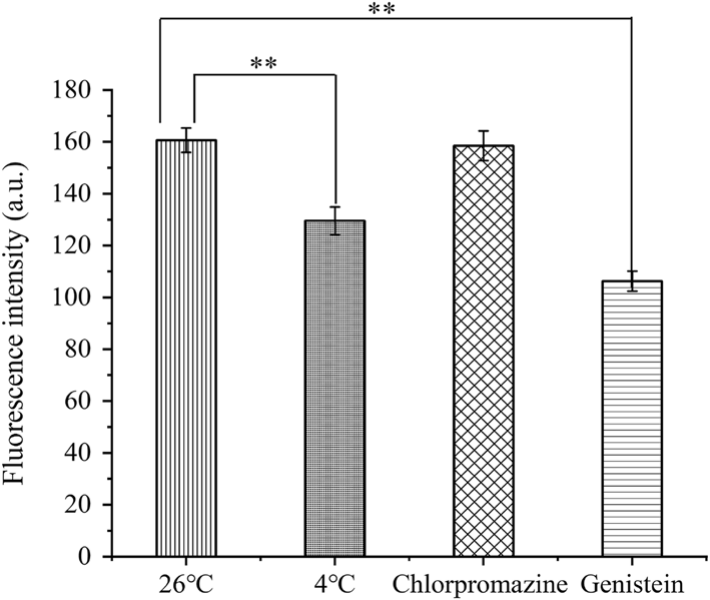
The fluorescence intensity inside yeast cells under different inhibition conditions(**P  < 0.01)

Yeast was incubated with BN-CDs for 8 h at 26 ℃, BN-CDs entered yeast cell smoothly (Fig. 8a), and the average fluorescence intensity in cells was 160.62 ± 4.67 a.u (Fig. 9). As we all know, low temperature will reduce the activity of intracellular enzymes, resulting in a decrease in mitochondrial energy production [55, 56]. After BN-CDs and yeast were incubated at 4 ℃ for 8 h, the fluorescence intensity in cells decreased significantly to 129.55 ± 5.32 a.u (P  < 0.05) (Fig. 9). The cellular uptake of BN-CDs at low temperature suggested that there were also non-energy dependent uptake pathways. Passive diffusion is a simple mode of transport without energy consumption. Ultra-small nanoparticles can enter cells through passive diffusion, such as metal nanoparticles less than 10 nm [57] and gadolinium nanoparticles less than 5 nm [58]. Therefore, it isuggested that the cellular uptake of BN-CDs (4 nm) was partially energy-dependent and passive diffusion also participated in the process. The mechanism of clathrin-mediated endocytosis is that extracellular macromolecules are packaged into clathrin cavities and taken up by cells in the form of clathrin-coated vesicles. Chlorpromazine is used to inhibit clathrin-mediated endocytosis by transferring clathrin and its connexin from the plasma membrane to endosomes, thereby inhibiting the formation of clathrin-coated cavities [59]. In the experiment, 200 μg/mL BN-CDs, 5 μg/mL chlorpromazine and yeast cells were incubated at 26 ℃ for 8 h. As shown in Fig. 8c, under the inhibition of chlorpromazine, the yeast still emitted bright blue light, and the average fluorescence intensity was 158.49 ± 5.69 a.u (P  > 0.05). It showed that endocytosis mediated by grid was not the main way for BN-CDs to enter yeast cell.

Caveolae is a small concave structure that exists on the surface of a variety of cells, and the small concave protein plays a central regulatory role in signal transduction [60]. Genistein is a tyrosine kinase inhibitor that can block caveolin-1 phosphorylation and is often used to inhibit caveolin-mediated endocytosis [60]. It can be seen from Fig. 8d that after 200 μg/mL BN-CDs, 40 μg/mL genistein and yeast cells were incubated at 26 ℃ for 8 h, the inside of yeast was obviously dark blue, and the cell wall and cytoplasm could be clearly distinguished. It was significantly different from the previous three components. Its average fluorescence intensity was 106.23 ± 3.87 a.u, which is significantly lower than the other three groups (Fig. 9). Therefore, it can be clarified that BN-CDs enter yeast cell mainly by caveolae mediated endocytosis.

## Conclusion

BN-CDs with stable fluorescence were synthesized by one-step microwave hydrothermal. Using BN-CDs to label yeast can clearly observe the growth status of yeast and accurately judge the division of yeast cell. Compared with other dyes, BN-CDs has low toxicity, simple labeling process and can quickly identify dead and living cells. The cellular uptake of BN-CDs is dose, time and partially energy dependent along with the involvement of passive diffusion. Caveolae mediated is the main mechanism of endocytosis.

## Experimental section

### Materials

All chemicals were at least of analytical reagent grade. Yeast peptone dextrose (YPD) medium was purchased from Qingdao Haibo Biological Co., Ltd. (Qingdao, China). Phosphate buffered saline (PBS), PI and FDA were supplied by Mclean Biochemical Technology Co. (Shanghai, China). Acetone, Congo red, chlorpromazine hydrochloride and genistein were provide by Tianjin Komeo Chemical Reagent Co., Ltd. (Tianjin, China). Citric acid, ethylenediamine, boric acid, ethanol, formaldehyde, and Meilan were purchased from Tianjin Kemei Chemical Reagent Co., Ltd. (Tianjin, China). Tobramycin sulfate was obtained from Shanghai Hefeng Pharmaceutical Co., Ltd. (Shanghai, China).

### Synthesis and characterization of BN-CDs

BN-CDs were prepared by microwave hydrothermal method as described by Ma et al.^[49]^with minor modifications. Citric acid (5 g), ethylenediamine (2 mL) and boric acid (2 g) were dissolved in 30 mL deionized water. The mixed solution was transferred to an autoclave, and the reactor was connected to a microwave-assisted synthesizer (XH-300A +, Beijing Xianhu Technology Development Co., Ltd., Beijing, China) to react at 200 ℃ for 1 h. Then the solution was cooled down to room temperature, and dialyzed with MWCO of 3500 Da for 24 h. The dialysate was concentrated by vacuum rotary evaporator, and the concentrated solution was lyophilized. Fluorescence spectra were obtained by an F-4500 spectrophotometer (Hitachi Ltd., Japan). Absorption spectra were recorded on a UV − vis spectrophotometer (UV-2550, Shimadzu Ltd., Japan). TEM measurements were performed on a model JEM-2100F transmission electron microscope (JEOL, Japan) for characterization of the shape and size of BN-CDs. XPS spectra were used to characterize the chemical composition using a K-Alpha X-ray Photoelectron Spectrometer (Thermo Fisher Scientific, USA).

### Measurement of Fluorescence QYs

The fluorescence QYs of BN-CDs was measured using quinine sulfate (dissolved in 0.1 M H_2_SO_4_, QYs  = 55%) as a standard and calculated using the following equation

$${\text{QY}} = {\text{Q}}_{{\text{R}}} \times \left( {{\text{I}}/{\text{I}}_{{\text{R}}} } \right)\left( {{\text{A}}_{{\text{R}}} /{\text{A}}} \right)\left( {{\text{n}}/{\text{n}}_{{\text{R}}} } \right)^{2}$$

where I is the measured fluorescence integral area, n shows the refractive index of the solvent, A denotes the absorbance, and subscript R is a known fluorescent standard substance.

### Yeast cell culture

The preserved yeast was inoculated into fresh YPD medium at an inoculum of 5% (v/v) and cultured in a constant temperature shaker at 26 ℃ and 100 r/min for 24 h. The culture medium was discarded, and the cells were washed three times with phosphate buffer for use.

### Observation on the growth status of yeast

BN-CDs (200 μg/mL) were incubated with yeast for 1 min, and the images of yeast in different growth status was observed with an objective lens of 100 × fluorescence microscope (Leica, German). In order to prove that BN-CDs staining can accurately identify the dead and living yeast cells, ultrasound, antibiotics, heating, microwave, ethanol, and formaldehyde were used to kill yeast, and the differences of yeast cell imaging between different lethal methods were compared.

### Cellular uptake kinetics

The yeast was activated and cultured in YPD medium for 24 h, then was washed three times with PBS (pH 7.2). It was incubated with BN-CDs (10, 50, 100, 150, 200, 300 μg/mL) for 2 h to determine the dose-dependent uptake. Yeast was incubated with 200 μg/mL BN-CDs for 1, 60, 120, 240, 360, 600 min, respectively. Three different visual fields were observed in each experiment, and the experiment was repeated three times. The average fluorescence intensity of living cells in 9 images was analyzed using Image J software [45].

### Cellular uptake pathways

The cellular energy-dependent uptake adopted a low-temperature treatment method. The yeast was pre-cooled at 4 ℃ for 1 h and then incubated with BN-CDs for 8 h. Different uptake inhibitors were used to determine the endocytosis mechanism of yeast.cell. Yeast was incubated with 5 μg/mL chlorpromazine and 40 μg/mL genistein at 26 ℃ for 1 h, then incubated with BN-CDs for 8 h. In the control group, BN-CDs were incubated with untreated yeast cells for 8 h. The fluorescence intensity in the cells was measured using a fluorescence microscope and Image J software.

## Supplementary Information

**Additional file 1: Figure S1.** Fluorescence lifetime spectrum of BN-CDs. **Figure S2.** The XPS full spectrum of BN-CDs. **Figure S3.** Cytotoxicity of BN-CDs on yeast cells.

## Data Availability

The authors confirm that the data supporting the findings of this study are available within the article.
