# Supplementary material for: B- and N-doped carbon dots by one-step microwave hydrothermal synthesis: tracking yeast status and imaging mechanism
Source: J Nanobiotechnology. 2021 Dec 28;19:456. doi: 10.1186/s12951-021-01211-w (PMC8715610; doi:10.1186/s12951-021-01211-w)
Supplement: Supplementary file 1 — Additional file 1: Figure S1. Fluorescence lifetime spectrum of BN-CDs. Figure S2. The XPS full spectrum of BN-CDs. Figure S3. Cytotoxicity of BN-CDs on yeast cells. [file 12951_2021_1211_MOESM1_ESM.docx]

**Supporting Information**

1. **and N-Doped Carbon Dots by One-Step Microwave Hydrothermal Synthesis: Tracking Yeast status and Imaging Mechanism**

Bo Tian^†^, Tianxin Fu^†^, Yang Wan^†^, Yun Ma^†^, Yanbo Wang^†^, Zhibiao Feng*^, ‡^, Zhanmei Jiang*^, †^

^†^College of Food Science, Northeast Agricultural University, Harbin,150030, China

^‡^ Department of Chemistry, Northeast Agricultural University, Harbin,150030, China

*Corresponding author. Tel.:+86-4515-519-0222

1. mail address: fengzhibiao@neau.edu.cn(Z. B. Feng)

zhanmeijiang@neau.edu.cn(Z. M. Jiang)

**1 Fluorescence lifetime spectrum of BN-CDs.**

As shown in Fig. S1, the average fluorescence lifetime of BN-CDs was 125.63 ns.

**
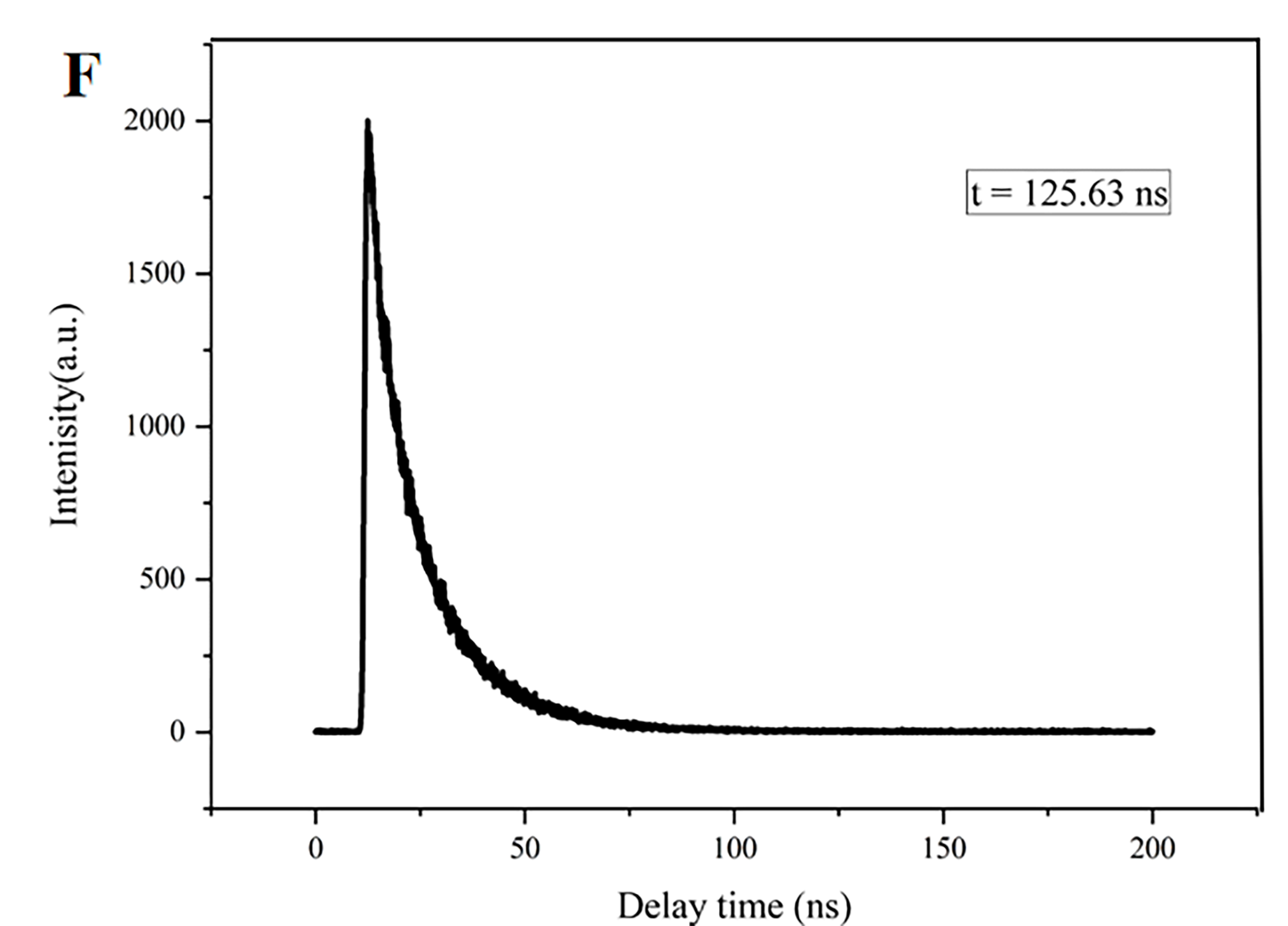
**

**Figure S1. Fluorescence lifetime of BN-CDs**

**2 The XPS full spectrum of BN-CDs**

**

**

**Figure S2. The XPS full spectrum of BN-CDs**

X-ray photoelectron spectroscopy (XPS) was used to further study the surface groups of BN CDs. The full spectrum shown in Fig. S2 shows four typical peaks: B 1s (191 eV), C 1s (284 eV), N 1s (398 eV) and O 1s (530 eV). It further proved that N and B were successfully doped into CDs.

**3 Cytotoxicity of BN-CDs on yeast.** The growth curve of yeast was plotted by growing yeast cells in 150 mL Erlenmeyer ﬂasks at 28 °C for 24 h. 0, 10, 20, 40, 100, 200, and 400 mg/mL of BN-CDs were added to Erlenmeyer ﬂasks and co-incubated by using orbital shaker at 180 rpm. The culture broth was collected every three hours and measured the optical density (at 600 nm) using a spectrophotometer (UV-2550, Shimadzu Ltd, Japan).





**Figure S3. Cytotoxicity of BN-CDs on yeast cells.**

The cytotoxicity of BN-CDs with different concentrations on yeast was showed in Figure S3. The result indicated that BN-CDs were non-toxic up to 400 mg/mL for yeast, which conﬁrmed that the BN-CDs could be used as eco-friendly biological ﬂuorescent-labeling probes in cellular imaging. It was found that 200 *μ*g/mL of BN-CDs was sufﬁcient for the tagging of multiple yeast. BN-CDs exhibited good biocompatibility and acted as nontoxic candidates for yeast imaging.
